# A Water-Soluble Chitosan Derivative for the Release of Bioactive Deferoxamine

**DOI:** 10.3390/ijms25020913

**Published:** 2024-01-11

**Authors:** Georgia Michailidou, Yupeng Li, Alexandra Zamboulis, Georgia Karlioti, Despoina Meimaroglou, Kostas Pantopoulos, Dimitrios N. Bikiaris

**Affiliations:** 1 Laboratory of Polymer and Colors Chemistry and Technology, Department of Chemistry, Aristotle University of Thessaloniki, 541 24 Thessaloniki, Greece; michailidougeorgia18@gmail.com (G.M.); azamboulis@gmail.com (A.Z.); geocar1997@gmail.com (G.K.); despinameimar@gmail.com (D.M.); 2 Department of Medicine, McGill University, Montreal, QC H3T 1E2, Canada; edward.li@mail.mcgill.ca; 3 Lady Davis Institute for Medical Research, Montreal, QC H3T 1E2, Canada

**Keywords:** deferoxamine, beta-thalassemia, chitosan, drug release, nanoparticles, solid dispersion, sustain release

## Abstract

Deferoxamine (DFO) is a water-soluble iron chelator used pharmacologically for the management of patients with transfusional iron overload. However, DFO is not cell-permeable and has a short plasma half-life, which necessitates lengthy parenteral administration with an infusion pump. We previously reported the synthesis of chitosan (CS) nanoparticles for sustained slow release of DFO. In the present study, we developed solid dispersions and nanoparticles of a carboxymethyl water-soluble chitosan derivative (CMCS) for improved DFO encapsulation and release. CS dispersions and nanoparticles with DFO have been prepared by ironical gelation using sodium triphosphate (TPP) and were examined for comparison purposes. The successful presence of DFO in CMCS polymeric dispersions and nanoparticles was confirmed through FTIR measurements. Furthermore, the formation of CMCS nanoparticles led to inclusion of DFO in an amorphous state, while dispersion of DFO in the polymeric matrix led to a decrease in its crystallinity according to X-ray diffraction (XRD) and differential scanning calorimetry (DSC) results. An in vitro release assay indicated sustained release of DFO from CS and CMCS nanoparticles over 48 h and 24 h, respectively. Application of CMCS-DFO dispersions to murine RAW 264.7 macrophages or human HeLa cervical carcinoma cells triggered cellular responses to iron deficiency. These were exemplified in the induction of the mRNA encoding transferrin receptor 1, the major iron uptake protein, and the suppression of ferritin, the iron storage protein. Our data indicate that CMCS-DFO nanoparticles release bioactive DFO that causes effective iron chelation in cultured cells.

## 1. Introduction

Thalassemia and sickle cell disease are common disorders in developing countries of tropical and subtropical regions, affecting more than 300,000 infants annually [1,2,3]. They are caused by inherited mutations in globin genes, which impair the appropriate production of hemoglobin (Hb), the oxygen transporter of red blood cells. It is estimated that 1–5% of the world population are carriers of thalassemia-causing mutation genes [4].

The clinical phenotype of the patients depends on the quantity of Hb production [1]. Patients with heterozygous globin gene mutations usually present with mild clinical symptoms and do not need treatment. However, homozygous patients require blood transfusion therapy for tissue oxygenation and survival, and to mitigate severe pathologies, such as growth retardation, bone deformities and hepatosplenomegaly [5]. One unit of transfused blood contains about 200–250 mg of iron. Because there is no iron excretion mechanism [6], excess iron is deposited in tissues, causing, among others, cardiomyopathy, liver disease, hypothyroidism, growth failure or delayed puberty [7]. In thalassemias and other iron-loading anemias with ineffective erythropoiesis (such as sideroblastic anemias, congenital dyserythropoietic anemias and some types of myelodysplastic syndromes), transfusional iron overload is also exacerbated by increased dietary iron absorption due to deregulation of the iron hormone hepcidin [8].

Iron chelation therapy was first introduced in the 1970’s with the clinical use of DFO, a hexadentate iron chelator and natural bacterial siderophore. With almost 50 years of experience, DFO remains an important therapeutic option. Patients receiving DFO are spared from major complications of transfusional siderosis and exhibit an extended life expectancy [9]. While DFO is water soluble and efficiently removes iron from plasma, liver and endocrine glands, it is poorly absorbed in the gastrointestinal tract and has a short plasma half-life of approximately 20 min. Thus, to reach effective pharmacological concentrations, the drug is administered parenterally with the aid of a portable infusion pump at least 4–5 days per week for 8–10 h each time. This cumbersome procedure reduces compliance and significantly compromises the quality of a patient’s life [10].

Encapsulation of DFO in nanocarriers such as biocompatible polymers for sustained controlled release would improve drug delivery and thereby offer unique therapeutic opportunities [11]. Consequently, the inclusion of DFO in various polymeric matrices has been examined. Our group has previously reported the preparation of CS-DFO-loaded nanoparticles [12]. Guo et al. [13] prepared mPEG-PLGA nanoparticles, and Vignesh et al. [14] developed PVA/PLGA nanoparticles in CS/hyaluronic acid hydrogel. Moreover, Li et al. [15] prepared cross-linked sodium alginate hydrogels containing DFO, while Zhu et al. developed conjugated polyphenol-DFO self-assembled nanoparticles [16]. All groups aimed at a more efficient and prolonged blood circulation time and indeed obtained encouraging results; nevertheless, there is still much room for improvement, especially regarding the entrapment efficiency of DFO.

Chitosan is a natural polysaccharide composed of glucosamine and N-acetylglucosamine monomers linked through β-(1−4)glycosidic bonds [17]. Chitin, the fully acetylated polymer, is derived from the exoskeleton of crustaceans and arthropods as well as from some fungi [18], while CS is obtained through its alkaline deacetylation. CS is the only positively charged natural polysaccharide and exhibits antibacterial properties and high solubility in acidic pH. However, it remains insoluble in physiological and alkaline environments [19]. The properties of CS can be potentially improved by chemical modifications. Quaternization of CS derivatives is a common procedure for water solubilization and carboxymethylation has emerged as a popular derivatization method, among various others described [20,21].

**Figure 1:**
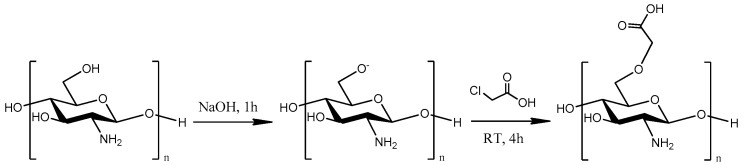
Synthetic route of CS modifications with monochloroacetic acid (MCAA).

In the present study, a water-soluble CS derivative, carboxymethyl CS (CMCS), was successfully synthesized (Figure 1). Two CMCS-based formulations with DFO were prepared: nanoparticles, obtained through ionic gelation, and solid dispersions. The successful incorporation of DFO into these formulations was evidenced and the properties of the newly formed formulations along with their in vitro release profile were examined. Corresponding formulations with neat CS were prepared as well for comparative purposes. The main objective was the preparation of an effective water-soluble nanocarrier for DFO which would improve in vitro release properties and biological activity.

## 2. Results and Discussion

### 2.1. Characterization of the CMCS Material

One of the main goals of the present study was the preparation of a water-soluble CS derivative. Modification of CS structures with MCAA has been explored by many research groups [22,23,24,25]. The molar ratio between CS and MCAA, the degree of the carboxylation and the carboxylation in N-, O- and N,O- positions are factors drastically affecting the formation of a water-soluble derivative. Successful modification of the CS structure was examined through FTIR measurements.

The typical absorption bands of CS in an IR spectrum are at 1656 and 1584 cm^−1^, which correspond to the vibration of the carbonyl bond C=O of the amide group and the angular deformation of the N-H bond, respectively. The absorption at 1418 cm^−1^ can be attributed to the same N-H group. The wide absorption band at 3400 cm^−1^ is essentially the result of overlapping absorptions due to O-H bond vibration, N-H bond vibration and polysaccharide hydrogen bonds. The absorbance at 1375 cm^−1^ is due to the symmetrical angular deformation of the C-H bond of the methyl acetyl groups, while that at 1324 cm^−1^ to the axial deformation of the C-N bond. Finally, the absorptions at 1074 and 1026 cm^−1^ are due to the secondary hydroxyl group (C-O bond vibration absorption in cyclic alcohols) and the primary hydroxyl group (C-O bond vibration absorption in primary alcohols, respectively) of the polysaccharide [17].

**Figure 2:**
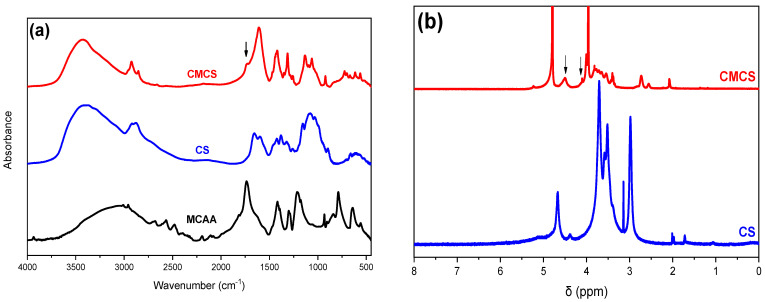
(**a**) FTIR spectra of MCAA, CS and CMCS and (**b**) 1H-NMR spectra of CS and CMCS.

The effective modification of a CS structure with MCAA is depicted in the FTIR spectrum of Figure 2a. The MCAA monomer exhibits a broad peak at 3040 cm^−1^, which corresponds to the vibration of the C-H bond while the signal displayed at 1736 cm^−1^ is due to the presence of the carbonyl group of the acid. In the CMCS spectrum, the characteristic new shoulder at 1730 cm^−1^ is attributed to the carbonyl groups inserted from MCAA in the CS structure. Furthermore, a new peak in the spectrum of the CMCS derivative appears, after the addition of the monomer. The signals at 1216 cm^−1^ and 1316 cm^−1^ are ascribed to the vibration of the C-O bond and to the carboxymethylation of both the amino and hydroxyl groups of the chitosan, respectively [22].

In agreement with FTIR, 1H-NMR measurements confirmed the successful modification of CS with MCAA. Figure 2b illustrates the spectrum of pure CS and the CMCS derivative. The existence of a peak at δ = 4.5 ppm and at δ = 4.09 ppm in the CMCS derivative is the indication of the successful derivatization since they correspond to the 6- and 3- protons of the O and N substitution, respectively, results which are according to the literature [24].

**Figure 3:**
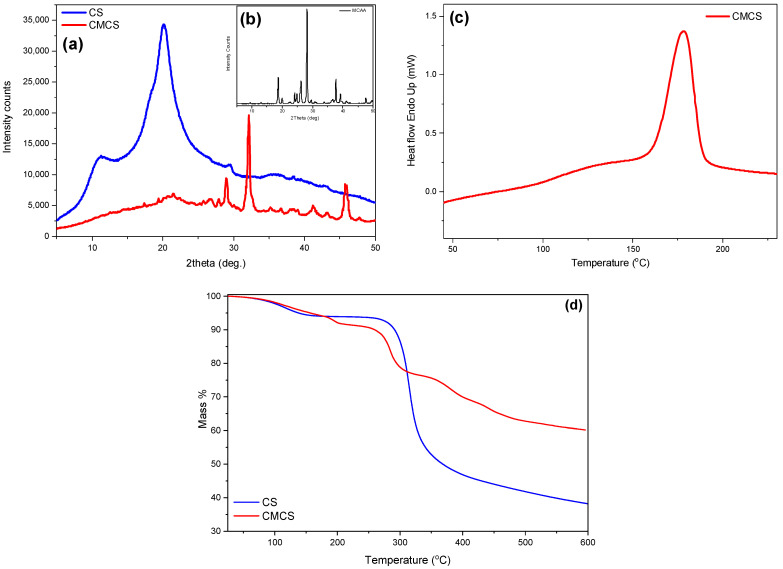
(**a**) XRD spectra of CS and CMCS, (**b**) XRD spectra of MCAA, (**c**) DSC thermogram of CMCS and (**d**)TGA thermogram of CS and CMCS.

The crystalline phase of CMCS was assessed through XRD measurements (Figure 3a). CS is a semicrystalline material exhibiting an amorphous region with two characteristic peaks at 2θ = 11° and 21° [26]. MCAA is a crystalline monomer with distinct characteristic crystalline peaks throughout its diffractogram. Regarding the CMCS derivative, it presents three acute crystalline peaks at 28.9°, 32.1° and 45.8°. Usually, modification of the CS structure results in an increased difficulty of the macromolecular chains to fold, leading to completely amorphous derivatives. However, CMCS presents crystalline peaks, revealing that the addition of the acetic acid group results in the formation of crystalline structures [27]. The preparation of a crystalline derivative is confirmed by DSC measurements. Typically, CS presents an endothermic peak at 70–90 °C, attributed to the dehydration of the material, while the degradation of CS is initiated after 300 °C [28]. The DSC thermogram of CMCS is presented in Figure 3c. The endothermic melting peak of CMCS present at 178 °C establishes the crystalline phase of the CMCS derivative.

Following analysis of the material’s crystalline phase and melting point, further thermal properties were examined through TGA measurements. TGA was recorded in order to examine the effect of the modification on the thermal stability of CS. The thermal decomposition of CS is described as a two-stage procedure. The first step between 50 and 150 °C corresponding to a mass loss of 6% is attributed to the loss of the unbound water, while the second step between 200 and 400 °C is associated with the degradation and deacetylation of CS [29]. CS continues to decompose above 400 °C, probably due to the degradation of the polysaccharide and the further decomposition of the chitosan molecules that did not degrade at lower temperatures [30]. At 600 °C, around 38% of the initial mass remained. Concerning the thermogram of CMCS, it is evident that the modification of the CS structure drastically affects the thermal behavior of the final material. In Figure 3d, a curve pattern with multiple mass loss stages is detected. The first step, at 50–150 °C according to CS, is attributed to the loss of the unbound water (5% of the total material’s mass). According to the literature, the second step, between 160 and 115 °C, and the third step, at 230–330 °C, are associated with the degradation of CS along with the decomposition of the MCAA monomer added on the CS backbone [31]. The initiation of the second step of CMCS thermal degradation is comparable to neat CS. However, the degradation of the macromolecular structure of CMCS occurs in multiple stages and the remaining mass residue is about 60%, significantly elevated in comparison to neat CS.

The solubility measurement of the new material was crucial since its solubility in neutral pH was a prerequisite. Table 1 presents the solubility values of CS and CMCS at pH 3, 7 and 11. CS is a freely soluble polymer in acidic pH; however, it remains practically insoluble in pH 7 and 11 with solubility percentages of 6.9 and 8.1, respectively [32]. CMCS is a freely soluble material in neutral and alkali pH and presents its lower solubility value in an acidic environment, results which are according to the literature [33]. The water solubility of CMCS is significantly improved in relation to the solubility of CS due to the introduction of the carboxymethyl groups. The presence of the COO^−^ groups on the CS structure are able to form hydrogen bonds with water molecules, rendering CMCS soluble in aqueous solutions [22]. The increased hydrophilic nature of CMCS is also established from the contact angle measurements presented on Table 1. CS has a high contact angle equal to 74.5°, revealing its low hydrophilicity [34]. The modification of the CS structure results in a lower contact angle measurement equal to 56.1°, establishing the increased hydrophilicity of CMCS.

### 2.2. Characterization of the DFO-Loaded Nanoparticles

The preparation of nanoparticles for both CS and CMCS materials was conducted through an ionic gelation technique using sodium tripolyphosphate (TPP). It is a simple technique for the preparation of nano and microparticles that is extensively applied for the encapsulation of various drugs and essential oils for further pharmaceutical or cosmetic applications [17,35,36,37,38]. The size, the polydispersity index (PdI) and the zeta potential of the prepared nanoparticles are provided in Table 2. The size of the CS nanoparticles lies between 194 and 340 nm, while the CMCS nanoparticles are between 205 and 335 nm with satisfying PdI. According to the literature, the size of the nanoparticles increases with increasing DFO content, while their PdI is also affected by the encapsulation, leading to higher dispersity values [39]. Nevertheless, concerning the CMCS nanoparticles, their size is decreasing with the increase in DFO content. This behavior is probably attributed to the hydrophilic character of both CMCS and DFO. The zeta potential for CS-TPP-DFO nanoparticles is higher than +30 mV, revealing the enhanced stability of the CS particles, while for CMCS-TPP-DFO the zeta potential is approximately +14 mV. The diminished value is expected because of the negative charge of the added carboxylic groups.

**Figure 4:**
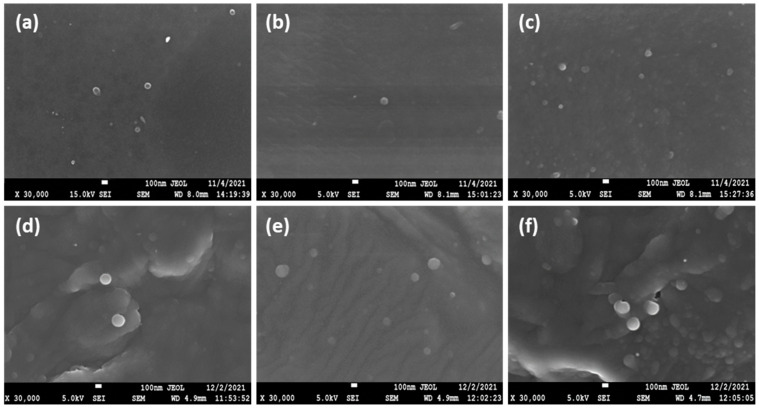
SEM images of (**a**) CS-TPP-DFO 25%, (**b**) CS-TPP-DFO 50%, (**c**) CS-TPP-DFO 75%, (**d**) CMCS-TPP-DFO 25%, (**e**) CMCS-TPP-DFO 50% and (**f**) CMCS-TPP-DFO 75%.

In a further step, SEM was utilized to examine the morphology of the prepared nanoparticles. As can be seen in Figure 4a–f, both CS and CMCS nanoparticles present a smooth surface with spherical morphology. SEM photos also revealed the size and polydispersity of the samples, as was also shown by DLS measurements. In accordance with the literature, the size of the particles depicted on SEM images appears to be lower than the size measured through DLS measurements, which is due to the presence of the dispersant [40,41].

**Figure 5:**
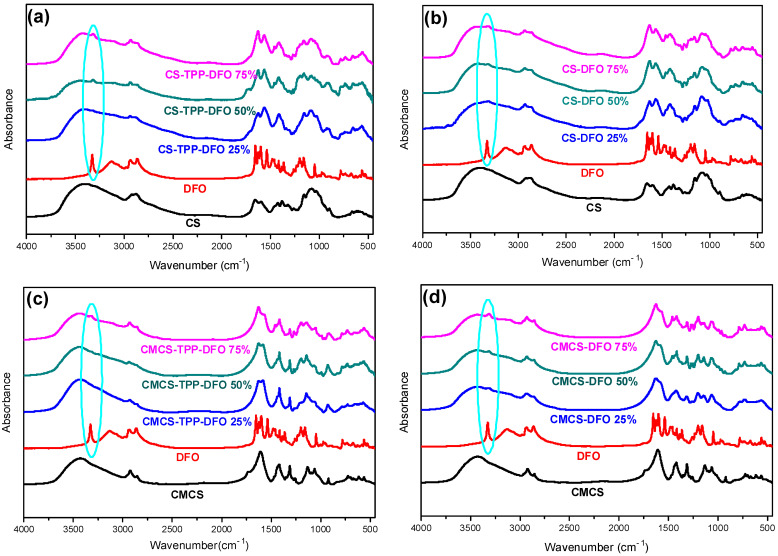
FTIR spectra of (**a**) CS, DFO and CS-TPP-DFO nanoparticles, (**b**) CS, DFO and CS-DFO solid dispersions, (**c**) CMCS, DFO and CMCS-TPP-DFO nanoparticles and (**d**) CMCS, DFO and CMCS-DFO solid dispersions.

The FTIR analysis was applied for the examination of the encapsulation of DFO in CS and CMCS nanoparticles as well as for the detection of any potential ionic interactions among the drug and the polymeric matrices in the nanoparticles and in the dispersions (Figure 5a–d). The typical DFO absorptions in an IR spectrum are present at 3423 cm^−1^ with a sharp band due to –OH stretching vibrations, at 3233 cm^−1^ with a broad peak that is assigned to N–H stretching vibrations and in the range of 1647–1596 cm^−1^, which is characteristic for the C=O bond and the N-H bend of the amino groups. The peak at 1474 cm^−1^ is attributed to the presence of the C=O portion of the aliphatic amides. Moreover, the presence of the C–N bond is evidenced by the peak at 1200 cm^−1^ while a small peak at 974 cm^−1^ indicates the N–O bend [42]. The characteristic hydroxyl, carbonyl and secondary amino groups on DFO structure could potentially interact with the hydroxyl, amino groups of CS and carboxylic groups of CMCS. According to Papadimitriou et al., in CS, nanoparticles prepared though ionic gelation technique with TPP, a shift of the characteristic amide bands of CS and an additional characteristic band at 910 cm^−1^ attributed to TPP are expected [43]. The FTIR spectra of CS-TPP-DFO nanoparticles is shown in Figure 5a. As can be seen, the peaks attributed to amide I and II of CS present at 1660 and 1595 cm^−1^ in neat CS spectrum are shifted to 1620 and 1560 cm^−1^ in the spectra of CS-TPP DFO samples, respectively, while a broad band of the phosphate groups of TPP is detected at 911 nm^−1^. Furthermore, it is evident that DFO was successfully incorporated into CS nanoparticles since a peak at 3320 cm^−1^ is present in the CS-TPP-DFO samples, attributed to the OH stretching vibration of DFO. Moreover, the peaks are slightly shifted to lower wave numbers, revealing the interaction of DFO with CS chains. Concerning the spectra of CMCS-TPP-DFO nanoparticles, the characteristic peak attributed to TPP is present at 925 cm^−1^, while the amide peaks of CMCS are present at 1628 and 1582 cm^−1^, respectively, revealing the interactions of CMCS with the TPP molecules. In a further step, the hydroxyl groups of DFO are present at 3324 cm^−1^, revealing the successful encapsulation of DFO in CMCS nanoparticles. Interestingly, the intensity of this particular peak increases with increasing DFO content. Concerning the CS and CMCS dispersions presented in Figure 5b,d, respectively, the slight shift of the amide peaks at 1629 and 1564 cm^−1^, along with the presence of the hydroxylic groups attributed to DFO at 3314 cm^−1^, indicate the presence of DFO along with the occurring interactions among DFO and the polymeric matrices.

**Figure 6:**
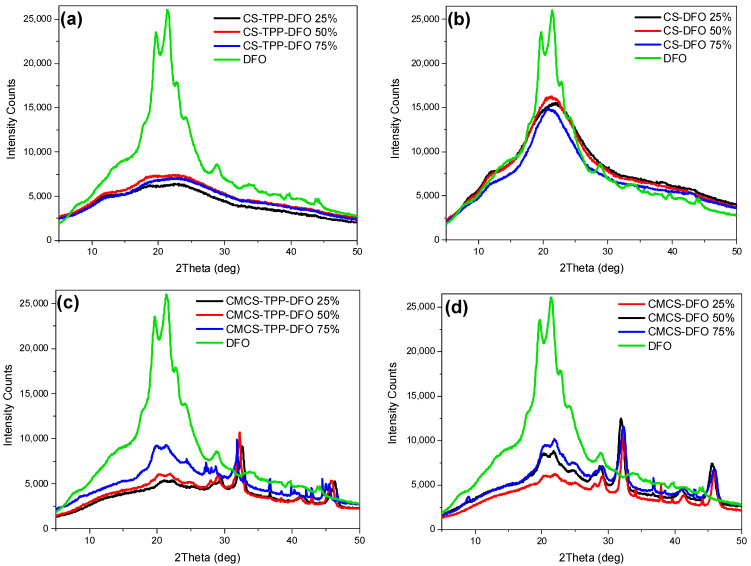
X-ray diffractograms of DFO and (**a**) CS-TPP-DFO nanoparticles, (**b**) CS-DFO solid dispersions, (**c**) CMCS-TPP-DFO nanoparticles and (**d**) CMCS-DFO solid dispersions.

In a subsequent stage, through the X-ray diffraction analysis, the physical state of the DFO-loaded nanoparticles and solid dispersions was estimated (Figure 6). DFO exhibits characteristic peaks at 2θ = 19.6°, 21.4°, 22.7°, 24.1° and 28.8°. Typically, when forming CS nanoparticles through the ionic gelation technique the polymeric chains are unable to fold, resulting in practically amorphous nanoparticles [44]. Concerning the CS nanoparticles, their X-ray diffractograms are presented in Figure 6a. It appears that DFO was successfully entrapped in an amorphous phase since the characteristic DFO peaks are absent. Concerning the CMCS nanoparticles (Figure 6c), DFO is entrapped in crystalline phase. The peaks at 19.9° and 21.4° are attributed to the crystalline phase of DFO, while the presence of other peaks in the CMCS-TPP-DFO diffractograms is ascribed to the crystalline nature of CMCS. Concerning the dispersions, preparation of amorphous solid dispersions through various techniques is a common approach for the amelioration of the bioavailability of crystalline drugs [45]. DFO in CS-DFO samples is dispersed in an amorphous state while the diffractograms of the samples resemble the diffractogram of neat CS since the chains are freely able to fold in the absence of TPP. In CMCS dispersions, a reduction in DFO crystallinity was observed. However, as in CMCS nanoparticle diffractograms, DFO crystalline peaks with reduced intensity are present at 20° and 22°, slightly shifted owing to interactions with the CMCS matrix. Moreover, the intensity of the crystalline peaks increases by increasing the percentage of DFO.

**Figure 7:**
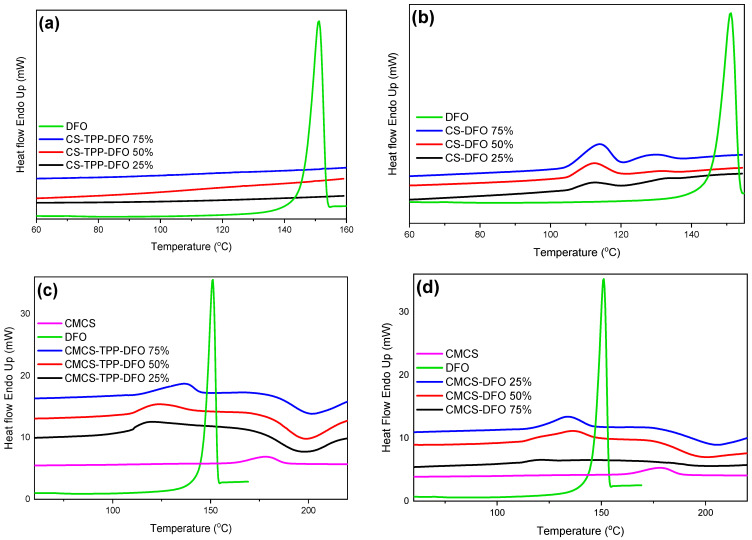
DSC thermograms of DFO and (**a**) CS-TPP-DFO nanoparticles, (**b**) CS-DFO solid dispersions, (**c**) CMCS-TPP-DFO nanoparticles and (**d**) CMCS-DFO solid dispersions.

The DSC thermograms of neat DFO and DFO nanoparticles and dispersions are presented in Figure 7. Neat DFO presents a melting peak at 151.2 °C. The amorphous character of the CS-TPP-DFO samples is confirmed through the DSC measurements. The melting peak of DFO is absent in the CS-TPP-DFO samples, indicating the amorphous character of the entrapped DFO. Regarding DFO dispersions in CS, we observe the presence of melting peaks at 120 °C, the size of which increases by increasing the amount of dispersed drug. The melting point is lower than the DFO melting point and this shift is due to the interaction of CS with DFO. This peak indicates that a small percentage of DFO is in the crystalline state. Nevertheless, these results are controversial since XRD data suggested that the CS-DFO dispersions were amorphous. According to the literature, when a very small number of crystallites are scattered within the amorphous matrix, the sensitivity of XRD might be insufficient to confirm the crystallinity even though, through DSC measurements, a crystallization peak is present [46]. Concerning the thermal diagrams of CMCS nanoparticles and DFO dispersion in CMCS, results confirm XRD diffractograms. Melting peaks are detected at 120 °C and 130 °C, respectively, attributed to the DFO crystals entrapped in crystalline form. Moreover, the melting peaks are shifted to higher temperatures and their intensities are increasing by increasing the amount of DFO present in the samples.

**Figure 8:**
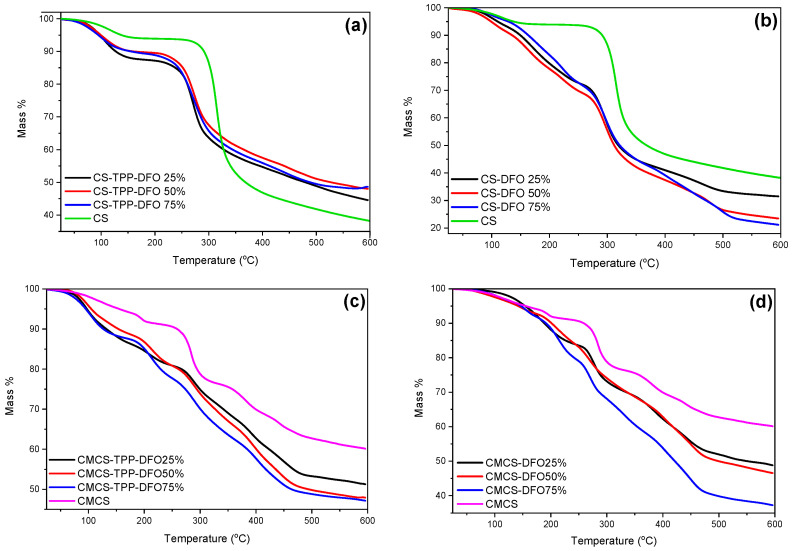
TGA thermograms of (**a**) CS and CS-TPP-DFO nanoparticles, (**b**) CS and CS-DFO solid dispersions, (**c**) CMCS and CMCS-TPP-DFO nanoparticles and (**d**) CMCS and CMCS-DFO solid dispersions.

Furthermore, the thermal stability of the nanoparticles and the solid dispersions were evaluated through TGA measurements. Concerning the thermogram of neat DFO, according to the literature, it presents two main degradation steps at 200 and 320 °C, corresponding to the decomposition of the organic compound [47]. CS nanoparticles (Figure 8a) present lower thermal stability in comparison to neat CS since the thermal degradation of their polymeric chains initiates at lower temperatures. This phenomenon is in accordance with previous data from our group where encapsulation of various active compounds resulted in diminished thermal stability [17]. Moreover, the remaining mass residue, varies depending on the DFO content, but is consistently higher in comparison to neat CS. It is attributed to the inorganic TPP crosslinker. Concerning the CS solid dispersions (Figure 8b), an additional mass loss step is observed starting at 250 °C. This step is attributed to possible interactions occurring among CS and DFO. Moreover, the mass residue is comparable to the CS since TPP is absent.

Regarding the samples of the materials synthesized with the modified chitosan (Figure 8c,d), they present a complex thermal pattern with multiple overlapped mass loss stages between the temperatures of 50 and 600 °C. The thermal stability of the CMCS nanoparticles and dispersions is diminished in comparison to neat CMCS, while the remaining mass residue is diminished and depends on the DFO content. Moreover, the presence of TPP in CMCS nanoparticles provokes higher mass residue in comparison to CMCS dispersions. It is evident that the synthesized nanoparticles and the dispersions reveal a mass loss pattern similar to the corresponding bulk materials. Furthermore, it is evident that the prepared samples are stable during storage.

### 2.3. In Vitro Release Studies

The drug-loading percentage of DFO in the prepared nanoparticles and solid dispersions is an important factor affecting the in vitro release profile of the drug along with its bioavailability [48]. Table 3 presents the drug-loading efficiency of the CS and CMCS nanoparticles and dispersions. Concerning the CS and CMCS nanoparticles, the drug loading is proportional to the amount of DFO added. DFO has already been encapsulated in various polymeric matrices. Its encapsulation in CS nanoparticles yielded results similar to ours [12,14], while when DFO was loaded in amphiphilic polymeric particles, enhanced loading capacity was achieved. More specifically, Qayoom et al. [49] loaded DFO in lecithin nanoparticles of various diameters and the loading percentages were up to 60% of the formulated nanoparticles. Moreover, DFO encapsulation in hydrophobic nanosized particles has been reported by many groups. Guo et al. [13] examined the effect of the ratio between synthetic polymer:DFO and showed that the lower polymer:DFO ratio resulted in lower encapsulation efficacy. In another study of our group, the inclusion of DFO in a hydrophobic polymeric matrix through the double emulsion technique resulted in low encapsulation efficacy since DFO remained in the aqueous phase while the polymers were dissolved in an organic phase. In the CMCS nanoparticles, the inclusion of the water-soluble DFO in a water-soluble polymeric matrix resulted in high drug-loading content. Similarly, regarding the CMCS solid dispersions, the drug content is proportional to the DFO quality used in the preparation of these formulations. This is attributed to the good solubility of CMCS in water. In contrast, in the CS dispersions, a random drug-loading efficacy is observed, which is attributed to the slightly hydrophobic nature of neat CS [50].

**Figure 9:**
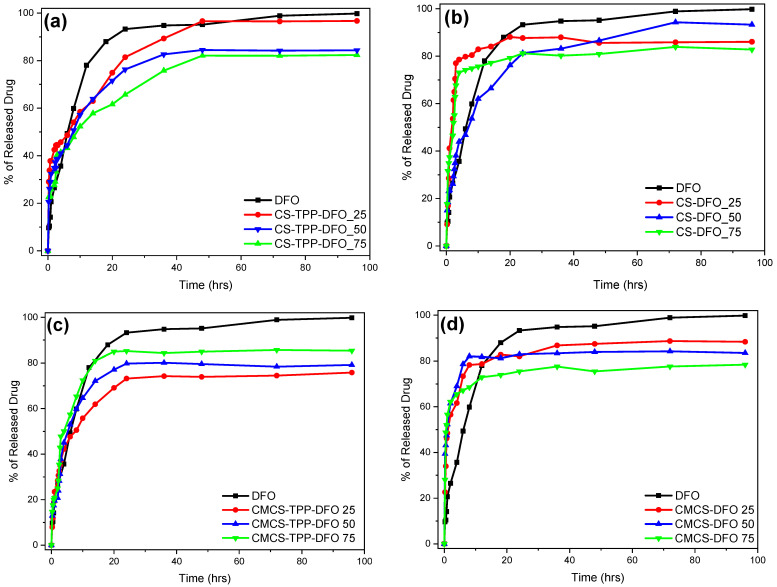
In vitro release rate of DFO from (**a**) CS-TPP-DFO nanoparticles, (**b**) CS-DFO solid dispersions, (**c**) CMCS-TPP-DFO nanoparticles and (**d**) CMCS-DFO solid dispersions.

The in vitro release behavior of the DFO-loaded samples is presented in Figure 9. DFO as a water-soluble active compound is characterized by a fast release profile with its dissolution reaching up to 93% during the first 24 h. In water-soluble drugs, a sustained release profile is preferred, which would potentially lead to a sustained in vitro release [51].

Several DFO in vitro release studies have been conducted by many research groups [52,53]. Ran et al. examined the in vitro release profile of DFO from Ti nanotubes and observed a burst release profile of 80% of the loaded DFO in almost 10 h [54]. In contrast, Marzban et al. [55] examined the DFO release from PEGylated nano-niosomals and after 20 h more than 80% of the encapsulated DFO remained in the particle’s interior, unable to be released. CS nanoparticles containing DFO have been previously examined by our group with exceptional results. Indeed, encapsulation of DFO in CS nanoparticles resulted in a more sustained release [12]. Nevertheless, when the release behavior of CS-TPP-DFO nanoparticles in cultured cells was studied, the release of DFO from CS nanoparticles was incomplete (unpublished work). This poor result was attributed to the low solubility of CS in a neutral environment. We therefore synthesized a more soluble CS derivative to increase the in vitro release, rationalizing that it would allow for better results in vivo. To further complete this study, the amelioration of the DFO dissolution profile by incorporation in solid dispersions was also examined.

Figure 9 presents the release profile of the CMCS nanoparticles performed at 37 ± 1 °C. For the sake of comparison and repeatability, DFO was also encapsulated in neat CS nanoparticles. The results obtained by CS-TPP-DFO are similar to our previous work [11]: an initial burst release during the first three hours followed by a sustained release up to 48 h. The DFO release of CS-TPP-DFO 25% reaches 97%, while the samples containing DFO in 50% and 75% released 84% and 81% of their drug content, respectively. As in CS nanoparticles, release of DFO from CMCS-TPP-DFO formulation occurs in two stages: an initial burst release during the first 4 h and a continuous release for a day. In other words, by encapsulating DFO in CMCS nanoparticles, the release of DFO was successfully accelerated compared to neat CS nanoparticles.

Furthermore, solid dispersions of CS and CMCS were prepared for optimization of DFO release [56]. However, the interactions between the polymeric matrices and DFO in the absence of TPP were insufficient for the maintenance of a sustained release behavior. Interestingly, dissolution of DFO in the CMCS dispersions is conducted at an even higher rate in comparison to neat DFO, this could be exploited in cases where an immediate DFO release for a fast action is required. Overall, a similar behavior is observed for the CS dispersions, with the exception of CS-DFO 50%. In conclusion, the inclusion of DFO in solid polymeric dispersions results in a fast and uncontrolled release attributed to the lack of sufficient interactions between DFO and CS or CMCS, especially taking into account its high solubility.

Overall, we can say that, as expected, CMCS nanoparticles accelerate the in vitro controlled release of DFO, and are thus promising candidates for in vivo experiments.

### 2.4. Biological Activity of DFO Released from CMCS-DFO

We assessed the biological activity of DFO released from CMCS-DFO in cell culture experiments. Treatment of cells with iron chelating drugs triggers homeostatic responses to iron deficiency, which are orchestrated by the IRE/IRP regulatory system [57]. In brief, iron deficiency activates “iron regulatory proteins” (IRP1 and IRP2) for binding to “iron responsive elements” (IREs) in the untranslated regions (UTRs) of mRNA’s encoding proteins of iron metabolism. These include, among others, transferrin receptor 1 (TFRC) and ferritin, which account for cellular iron uptake and storage, respectively. The binding of IRPs to IREs in the 3′ UTR of TFRC mRNA stabilizes it against nucleolytic degradation, thereby increasing its expression. Conversely, the binding of IRPs to an IRE in the 5′ UTR of FTH and FTL mRNAs (encoding ferritin H- and L-subunits, respectively) inhibits their translation, thereby reducing cellular ferritin content. These responses program the cells to enhance iron uptake for metabolic purposes and prevent its storage when the metal is scarce.

**Figure 10:**
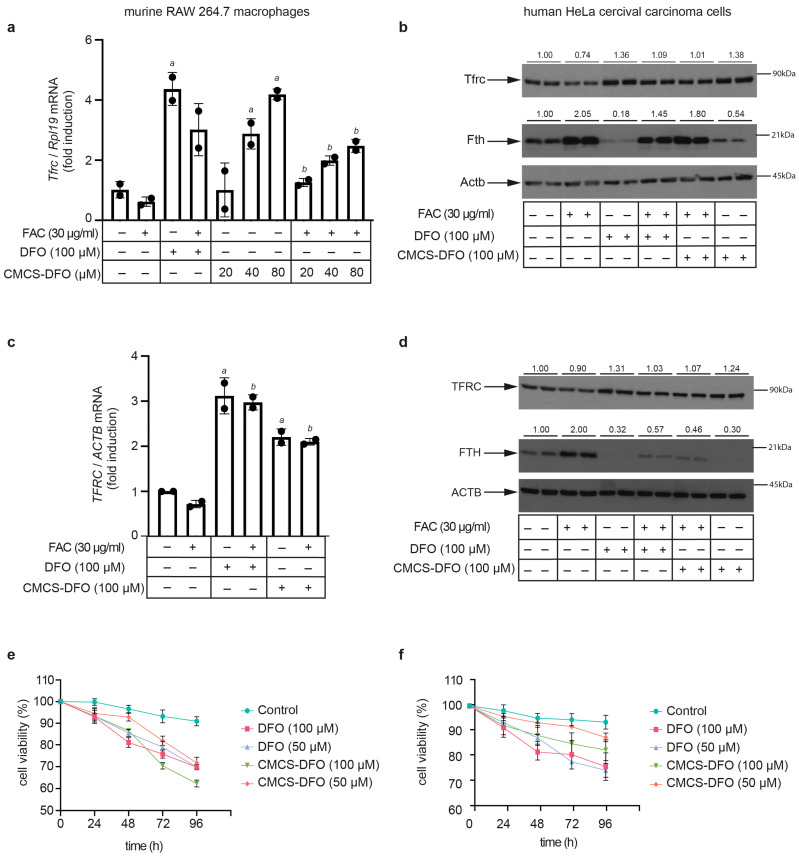
CMCS-DFO dispersions release bioactive DFO that promotes iron deficiency responses in cell-culture models. RAW 264.7 macrophages or HeLa cells were either left untreated or pretreated for 24 h with 30 μg/mL ferric ammonium citrate (FAC). Subsequently, the cells were washed and further incubated for 18 h (**a**–**d**) or for 24–96 h (**e**,**f**) without or with various doses of free DFO or DFO released from CMCS-DFO 50%. The concentrations of DFO released from CMCS-DFO 50% were calculated assuming 100% release efficiency; a stock solution was prepared by dissolving 0.05 g CMCS-DFO 50% in 2 mL Milli-Q ultrapure water. (**a**) qPCR analysis of Tfrc mRNA in RAW 264.7 macrophages; (**b**) Western blot analysis of Tfrc (top), Fth (middle) and Actb (bottom) in RAW 264.7 macrophages; (**c**) qPCR analysis of TFRC mRNA in HeLa cells; (**b**) Western blot analysis of TFRC (top), FTH (middle) and ACTB (bottom) in HeLa cells; (**e**,**f**) kinetic analysis of the effects of free DFO and CMCS-DFO 50% on viability of RAW 264.7 macrophages (**e**) and HeLa cells (**f**); cell viability was assessed by the trypan blue exclusion assay. The qPCR data in (**a**,**c**) are presented as geometric mean ± geometric standard deviation. Statistically significant differences were identified by the Student’s *t* test (a denotes *p* ≤ 0.05 vs. untreated control and b denotes *p* ≤ 0.05 vs. FAC-pretreated). Immunoreactive bands in (**b**–**d**) were quantified by densitometry; the ratios to β-actin are shown on top.

In preliminary experiments, murine RAW 264.7 macrophages were treated with various preparations of CS-DFO and CMCS-DFO nanoparticles and dispersions. A dispersion of CMCS-DFO 50% triggered consistent and potent induction of Tfrc mRNA and was therefore selected for further characterization. To this end, RAW 264.7 cells were left untreated, or were previously subjected to iron loading with ferric ammonium citrate (FAC). Subsequently, untreated or FAC pre-treated cells were exposed to free DFO or to increasing doses of CMCS-DFO 50% for 18 h. As expected, the FAC pre-treatment decreased Tfrc mRNA levels (Figure 10a). Importantly, the CMCS-DFO dispersion induced Tfrc mRNA in both control and FAC-pretreated cells in a dose-dependent manner. Thus, CMCS-DFO exhibited similar activity to free DFO. In another experiment with RAW 264.7 cells, we evaluated the effects of free DFO and CMCS-DFO on Tfrc and ferritin protein levels by Western blotting. Again, both CMCS-DFO and free DFO effectively induced Tfrc in control cells, and rescued Tfrc expression in FAC-pretreated cells (Figure 10b, top panel). Moreover, both CMCS-DFO and free DFO suppressed Fth (H-ferritin) expression, even following its previous induction by FAC (Figure 10b, middle panel). Levels of housekeeping Actb (β-actin) did not change during iron perturbations (Figure 10b, bottom panel), indicating equal loading. Similar results were obtained in experiments with human HeLa cervical carcinoma cells (Figure 10c,d), demonstrating that the biological activity of CMCS-DFO is not species- or cell-specific.

Prolonged exposure of cells to iron chelating drugs is known to cause growth arrest and apoptosis [58]. Along these lines, CMCS-DFO and free DFO similarly decreased the viability of RAW 264.7 (Figure 10e) and HeLa cells (Figure 10f) over 96 h. Significant decreases in cell viability were already registered at 24 h, which again indicates effective release of DFO from CMCS-DFO and is consistent with the results in Figure 9. Overall, our data demonstrate that the CMCS-DFO dispersion releases bioactive DFO that can chelate iron and neutralize the effects of iron overload in cultured cells.

## 3. Materials and Methods

### 3.1. Materials and Reagents

Chitosan was supplied by Kraeber and Co GmbH (Ellerbek, Germany), possessing a molecular weight of 18,000 g/mol and a degree of deacetylation > 94%, as was determined by viscometry and 1H NMR, respectively, in our previous study [17]. Monochloroacetic acid was supplied by Alfa-Aesar (Kandel, Germany). Sodium tripolyphosphate (TPP) used as ionic crosslinker (85% purity) and acetonitrile HLPC purity (ACN) were supplied from Aldrich chemicals (Steinheim, Germany). Deferoxamine was kindly donated by Mayne Pharma Inc. (Montreal, Canada). All other reagents used were of analytical grade.

### 3.2. Synthesis of CMCS Derivative

The synthesis of the CS derivative was performed (Figure 1) according to Bidgoli et al. [33]. Briefly, 17 g of NaOH were dissolved in water (20 mL). CS (10 g) and 2-propanol (80 mL) were added in the NaOH solution and CS was swollen and alkalized under magnetic stirring for 1 h. Then, 17 g of monochloroacetic acid (MCAA) were dissolved in 30 mL of 2-propanol and the resulting mixture was added dropwise to the alkalized CS mixture. Thereafter, the CS suspension was stirred for 4 h. The end of the reaction occurred by the addition of EtOH. The carboxymethyl-CS (CMCS) product was retrieved by vacuum filtration and washed several times with EtOH. Soxhlet extraction was performed for the removal of by-products or any unreacted monomer using EtOH solvent.

### 3.3. Preparation of Nanoparticles

CS nanoparticles were prepared according to a well-established ionotropic gelation technique [43]. Briefly, the proper amount of CS was dissolved in a 25 mL aqueous solution of acetic acid 2% *v*/*v* (pH 4.5), forming a solution 0.8% *w*/*v*. The proper amount of DFO was added to the CS solution in final concentrations of 25 wt%, 50 wt% and 75 wt% in DFO to the CS polymer matrix, followed by magnetic stirring for 30 min and probe sonication (100 W, 30 kHz, Hielscher Ultrasonics, Teltow, Germany) for 2 min. An aqueous solution of TPP, 2 mg/mL in concentration and 25 mL in volume, was inserted dropwise to the CS-DFO solutions, under magnetic stirring. According to previous studies from our group, there is a critical ratio between CS and TPP where the nanoparticles are produced with the smallest size. As a result, the ratio of CS/TPP was 4/1. The nanoparticles were stirred for 4 h and centrifuged at 11,000 rpm for 20 min (Heraeus™ Pico™ 17 Microcentrifuge, Thermo Fisher Scientific, Waltham, MA, USA), washed with water and resuspended in water. After a freeze-drying procedure, nanoparticles were kept in vacuum for further use.

CMCS nanoparticles were prepared according to the procedure described above with the dissolution of the CMCS material occurring in distilled water. The pH of the polymeric solution was adjusted to 5.6.

### 3.4. Preparation of Solid Dispersions

For the preparation of solid dispersions, a slightly modified solvent evaporation technique was applied. Briefly, the proper amount of CS was dissolved in 25 mL CH_3_COOH 2% *v*/*v*, forming a polymeric solution of 0.8% *w*/*v*. The proper amount of DFO was added in the polymeric solutions, creating final samples containing 25 wt%, 50 wt% and 75 wt% in DFO to polymeric matrix. The samples were under magnetic stirring for 30 min and probe sonication for 2 min. The samples were frozen, freeze-dried to remove the solvent and subsequently were kept in vacuum for further use.

CMCS dispersions were prepared according to the procedure described above with the dissolution of the CMCS material occurring in distilled water.

### 3.5. Characterization of Materials and DFO-Loaded Formulations

#### 3.5.1. Fourier-Transformed Infrared Spectroscopy (FTIR)

FTIR spectra of the samples were obtained on a Perkin Elmer FTIR spectrometer (model FTIR-2000, Perkin Elmer, Waltham, MA, USA). Each sample was triturated with a proper amount of potassium bromide (KBr) and the disks were formed under pressure. The spectra were collected in the range of 400 to 4000 cm^−1^ at a resolution of 4 cm^−1^ using 16 co-added scans and the baseline was corrected and converted into absorbance mode.

#### 3.5.2. Wide-Angle X-ray Scattering (XRD)

X-ray powder diffraction (XRD) patterns were recorded using an XRD-diffractometer (Rigaku Co. MiniFlex II XRD system, Oxford, UK) with a CuKα radiation for crystalline phase identification (λ = 0.15405 nm). The sample was scanned at the range of 5 to 50° with a scan speed of 1 °/min.

#### 3.5.3. Dynamic Light Scattering (DLS)

The size of the nanoparticles was determined using dynamic light scattering (Zetasizer 5000, Malvern company, Worcestershire, UK). Suspended nanoparticles of 100 μL were dispersed in 900 μL of double-distilled water. All measurements were performed in triplicate.

#### 3.5.4. Differential Scanning Calorimetry (DSC)

For differential scanning calorimetry analysis, a Perkin–Elmer Pyris 1 differential scanning calorimeter (DSC) (Waltham, MA, USA), calibrated with Indium and Zinc standards, was used. About 10 mg of each sample was used, placed in a sealed aluminum pan and heated up from 30 to 105 °C with a heating rate of 20 °C/min in an inert atmosphere (N2, flow rate 50 mL/min), held in 105 °C for 1 min in order to remove the absorbed water, cooled to 30 °C with a cooling rate of 20 °C/min and heated up again from 30 to 240 °C. The data reported in this work were acquired from the second heating scan.

#### 3.5.5. Thermogravimetric Analysis (TGA)

Thermogravimetric analysis (TGA) was conducted in a Labsys evo TG/DSC 1150 instrument (Setaram Instrumentation, Lyon, France). Samples of 3 ± 0.5 mg were placed in alumina pans. An empty alumina pan was used as a reference. Samples were dried overnight at 60 °C to remove the absorbed moisture. Heating was controlled by rotating temperature from RT up to 600 °C in a 50 mL/min flow of N2. The heating rate was set at 20 °C/min and steady marks of sample temperature, sample weight, and heat flow were recorded.

#### 3.5.6. Scanning Electron Microscopy (SEM)

SEM images were acquired with a high-resolution electron microscope JSM-7610F (Akishima, Tokyo, Japan). A drop of each nanoparticles’ suspension was placed on the holder and left to evaporate. Samples were covered with carbon to provide a good conductivity of the electron beam. Operating conditions were set at accelerating voltage 20 kV, probe current 45 nA and counting time 60 s.

#### 3.5.7. Contact Angle

For the calculation of contact angles, films of approximately 1 × 1 cm^2^, prepared by solvent evaporation at 60 °C, were placed onto the microscope glass. Contact angles were measured in water, employing the sessile drop method with Ossila Contact Angle Goniometer L2004A1 (Ossila Ltd., Shiefield, UK). The experiment was performed in triplicate. The results were expressed as mean ± standard deviation (SD).

#### 3.5.8. High-Pressure Liquid Chromatography (HPLC)

Quantitative analysis and drug-loading quantitative analysis were performed using a Shimadzu HPLC (Kyoto, Japan) prominence system consisting of a degasser (DGU-20A5, Kyoto, Japan), a liquid chromatograph (LC-20 AD, Kyoto, Japan), an autosampler (SIL-20AC, Kyoto, Japan), a UV/Vis detector (SPD-20A, Kyoto, Japan) and a column oven (CTO-20AC, Kyoto, Japan). For the analysis, the well-established method of You et al. was used [59]. In detail, CNW Technologies Athena C18, 120 A, 5 μm, 250 mm × 4.6 mm at a column temperature of 25 °C. The mobile phase consisted of ACN/H2O 80/20 *v*/*v*, at a flow rate of 1.0 mL/min. The injection volume was 20 μL and UV detection was performed at 430 nm at 25 °C. The calibration curve was created by diluting a stock methanol solution of 100 ppm DFO to concentrations of 0.01, 0.05, 0.1, 0.25, 0.5, 1.0, 2.5, 5.0, 10.0, 20.0, 30.0 and 50.0 ppm using ultrapure water. For the determination of the drug-loading capacity of the nanoparticles, 10 mg of the prepared nanoparticles were dissolved in 10 mL of a mixture of aqueous acetic acid solution (1% *v*/*v*): methanol (50:50 *v*/*v*). The resulting solutions were stirred for 24 h and filtered (nylon filters, 0.45 nm pore size).

#### 3.5.9. In Vitro Dissolution Studies

For the in vitro release studies, DISTEK Dissolution Apparatus I (North Brunswick, NJ, USA), equipped with an autosampler, was used. Nanoparticles were inserted in dialysis tubing, (molecular weight cut-off 12,000–14,000, Servapor) and placed in the baskets of the apparatus. Dissolution was performed at 37 ± 1 °C and the rotation speed was set at 50 rpm. The dissolution medium was 300 mL of a phosphate buffer, pH = 7.4. At interval times, two milliliters of aqueous solution were withdrawn from the release media and quantified.

#### 3.5.10. Cell Culture

Murine RAW 264.7 macrophages or human HeLa cervical carcinoma cells were grown at 37 °C in a humidified incubator in the presence of 5% CO_2_ using Dulbecco’s modified Eagle medium (DMEM) supplemented with 10% fetal bovine serum, 100 U/mL penicillin and 100 mg/mL streptomycin (Wisent Inc., St-Bruno, QC, Canada). At the experimental endpoints, the cells were scraped, harvested, washed in cold phosphate buffered saline (PBS) and separated into two fractions for subsequent analysis by qPCR or Western blotting.

#### 3.5.11. Quantitative Real-Time PCR (qPCR)

Cell pellets were lysed in RLT Buffer. Total RNA was isolated from cell lysates using the RNeasy Mini kit (Qiagen). Purity was assessed by 260/280 nm absorbance ratios. qPCR was performed by using species- and gene-specific primers (Table 4), as previously described [60]. Data were normalized to murine Rpl19 for RAW 264.7 macrophages and human ACTB for HeLa cells. Relative mRNA expression was calculated by the comparative Ct method. qPCR results are represented as fold changes compared to untreated control cells.

#### 3.5.12. Western Blotting

Cell pellets were lysed in a buffer containing 1% Triton X-100, 25 mM Tris–Cl, pH 7.4 and 40 mM KCl, as previously described [61]. Cell lysates containing 30 μg of proteins were analyzed by SDS-PAGE on 10% or 15% gels, and proteins were transferred onto nitrocellulose membranes (BioRad, Hercules, CA, USA). The blots were blocked in 10% bovine serum albumin or 10% fat-free skim milk in tris-buffered saline (TBS), containing 0.1% (*v*/*v*) Tween-20 (TBS-T), and probed overnight with primary antibodies against transferrin receptor 1 (TFRC from Zymed; mouse monoclonal; 1:1000 diluted), H-ferritin (FTH from Novus Biologicals; rabbit polyclonal; 1:500 diluted) or β-actin (ACTG from Sigma-Aldrich; rabbit polyclonal; 1:1000 diluted). Following a 3x wash with TBS-T, the membranes were incubated with 1:5000-diluted anti-mouse or 1:20,000 diluted anti-rabbit peroxidase-coupled secondary antibodies for 2 h. Immunoreactive bands were detected by enhanced chemiluminescence with the Western Lightning ECL Kit (Perkin Elmer, Shelton, CT, USA).

#### 3.5.13. Cell Viability

The impact of CMCS-DFO treatments on cell viability was assessed with the Trypan blue exclusion assay [57].

## 4. Conclusions

In the present study, potential nanocarriers for the ameliorated and prolonged in vitro release of DFO were developed and characterized. A water-soluble derivative of CS was synthesized through modification of the CS structure with carboxymethyl groups. The successful synthesis was confirmed through FTIR and ^1^H-NMR measurements, while the CMCS material presented high solubility in both neutral and alkaline environments. As DFO nanocarriers, CS and CMCS, both solid dispersions and nanoparticles, were examined. Interactions between the drug and the polymeric matrices were examined through FTIR, while the crystalline and thermal properties of the DFO-loaded samples were assessed through XRD and DSC measurements. The crystallinity of DFO was diminished in both the dispersions and nanoparticles. The interactions occurring between the polymeric matrix and the drug in the solid dispersions of CS and CMCS materials were weak and a fast in vitro dissolution profile of DFO was achieved, while a sustained and prolonged in vitro release from CS and CMCS nanoparticles was attained for 48 and 24 h, respectively. Importantly, the CMCS-DFO dispersion exhibited high biological activity in two different cell culture models of murine and human origin, which was comparable to that of free DFO. Our data suggest that these nanoparticles are promising candidates for the treatment of patients with transfusional iron overload; however, further validation studies using in vitro cell culture and in vivo animal models are required.

## Figures and Tables

**Table 1:** Percentage (%) of solubility of CS and CMCS at pH 3, 7 and 10 and contact angle measurements of CS and CMCS.

	Solubility (%)			Contact Angle, θ (°)	
	pH = 3	pH = 7	pH = 10		
**CS**	100	6.9	8.1	74.5	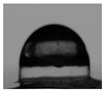
**CMCS**	92.4	95	97.5	56.1	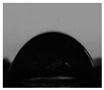

**Table 2:** Size, PdI and zeta potential values of CS and CMCS nanoparticles.

Sample Name	Z-Average (d.nm)	PdI	Zeta Potential (mV)
**CS-TPP-DFO 25%**	194	0.227	33.7
**CS-TPP-DFO 50%**	210	0.185	30.8
**CS-TPP-DFO 75%**	340	0.348	32
**CMCS-TPP-DFO 25%**	335	0.561	14.9
**CMCS-TPP-DFO 50%**	265	0.580	14.6
**CMCS-TPP-DFO 75%**	205	0.581	14.8

**Table 3:** DFO nanoparticle and solid dispersion drug-loading percentages.

Sample	Drug Loading (%)
CS-TPP-DFO 25% CS-TPP-DFO 50% CS-TPP-DFO 75%	24.13 27.01 56.64
CS-DFO 25% CS-DFO 50% CS-DFO 75%	41.67 16.13 31.19
CMCS-TPP-DFO 25% CMCS-TPP-DFO 50% CMCS-TPP-DFO 75%	30.00 66.95 92.20
CMCS-DFO 25% CMCS-DFO 50% CMCS-DFO 75%	38.35 42.56 67.45

**Table 4:** List of primers used for qPCR.

Gene	GenBank Accession No	Forward Primer Sequence	Reverse Primer Sequence
*Tfrc*	NM_011638.4	AGCCAGATCAGCATTCTCTAACT	GCCTTCATGTTATTGTCGGCAT
*Rpl19*	NM_009078.2	AGGCATATGGGCATAGGGAAGAG	TTGACCTTCAGGTACAGGCTGTG
*TFRC*	NM_003234	GCAAGTAGATGGCGATAACAG	GACGATCACAGCAATAGTCCC
*ACTB*	NM_001101.3	AGGATGCAGAAGGAGATCACT	GGGTGTAACGCAACTAAGTCATAG

## Data Availability

Data are contained within the article.

## References

[1] Chauhan W., Shoaib S., Fatma R., Zaka-ur-Rab Z., Afzal M. (2022). β-thalassemia, and the advent of new Interventions beyond Transfusion and Iron chelation. Br. J. Clin. Pharmacol..

[2] Piel F.B., Steinberg M.H., Rees D.C. (2017). Sickle cell disease. N. Engl. J. Med..

[3] Kattamis A., Kwiatkowski J.L., Aydinok Y. (2022). Thalassaemia. Lancet.

[4] De Simone G., Quattrocchi A., Mancini B., di Masi A., Nervi C., Ascenzi P. (2022). Thalassemias: From gene to therapy. Mol. Asp. Med..

[5] Fucharoen S., Ketvichit P., Pootrakul P., Siritanaratkul N., Piankijagum A., Wasi P. (2000). Clinical manifestation of β-thalassemia/hemoglobin e disease. Am. J. Pediatr. Hematol. Oncol..

[6] Fibach E., Rachmilewitz E.A. (2017). Pathophysiology and treatment of patients with beta-thalassemia—An update. F1000Research.

[7] Khandros E., Kwiatkowski J.L. (2019). Beta Thalassemia: Monitoring and New Treatment Approaches. Hematol. Oncol. Clin. N. Am..

[8] Cazzola M. (2022). Ineffective erythropoiesis and its treatment. Blood.

[9] Brittenham G.M., Griffith P.M., Nienhuis A.W., McLaren C.E., Young N.S., Tucker E.E., Allen C.J., Farrell D.E., Harris J.W. (1994). Efficacy of Deferoxamine in Preventing Complications of Iron Overload in Patients with Thalassemia Major. N. Engl. J. Med..

[10] Reddy P.S., Locke M., Badawy S.M. (2022). A systematic review of adherence to iron chelation therapy among children and adolescents with thalassemia. Ann. Med..

[11] Jones G., Goswami S.K., Kang H., Choi H.S., Kim J. (2020). Combating iron overload: A case for deferoxamine-based nanochelators. Nanomedicine.

[12] Lazaridou M., Christodoulou E., Nerantzaki M., Kostoglou M., Lambropoulou D.A., Katsarou A., Pantopoulos K., Bikiaris D.N. (2020). Formulation and in-vitro characterization of chitosan-nanoparticles loaded with the iron chelator deferoxamine mesylate (DFO). Pharmaceutics.

[13] Guo S., Liu G., Frazer D.M., Liu T., You L., Xu J., Wang Y., Anderson G.J., Nie G. (2018). Polymeric Nanoparticles Enhance the Ability of Deferoxamine to Deplete Hepatic and Systemic Iron. Nano Lett..

[14] Vignesh S., Sivashanmugam A., Annapoorna M., Janarthanan R., Subramania I., Shantikumar V.N., Jayakumar R. (2018). Injectable deferoxamine nanoparticles loaded chitosan-hyaluronic acid coacervate hydrogel for therapeutic angiogenesis. Colloids Surf. B Biointerfaces.

[15] Li S., Wang X., Chen J., Guo J., Yuan M., Wan G., Yan C., Li W., Machens H.G., Rinkevich Y. (2022). Calcium ion cross-linked sodium alginate hydrogels containing deferoxamine and copper nanoparticles for diabetic wound healing. Int. J. Biol. Macromol..

[16] Zhu F., Zhong J., Hu J., Yang P., Zhang J., Zhang M., Li Y., Gu Z. (2022). Carrier-Free Deferoxamine Nanoparticles against Iron Overload in Brain. CCS Chem..

[17] Michailidou G., Ainali N.M., Xanthopoulou E., Nanaki S., Kostoglou M., Koukaras E.N., Bikiaris D.N. (2020). Effect of Poly(vinyl alcohol) on Nanoencapsulation of Budesonide in Chitosan Nanoparticles via Ionic Gelation and Its Improved Bioavailability. Polymers.

[18] Sahariah P., Másson M. (2017). Antimicrobial Chitosan and Chitosan Derivatives: A Review of the Structure-Activity Relationship. Biomacromolecules.

[19] Zamboulis A., Nanaki S., Michailidou G., Koumentakou I., Lazaridou M., Ainali N.M., Xanthopoulou E., Bikiaris D.N. (2020). Chitosan and its derivatives for ocular delivery formulations: Recent advances and developments. Polymers.

[20] le Dung P., Milas M., Rinaudo M., Desbrières J. (1994). Water soluble derivatives obtained by controlled chemical modifications of chitosan. Carbohydr. Polym..

[21] Jimtaisong A., Saewan N. (2014). Utilization of carboxymethyl chitosan in cosmetics. Int. J. Cosmet. Sci..

[22] Chen X.G., Park H.J. (2003). Chemical characteristics of O-carboxymethyl chitosans related to the preparation conditions. Carbohydr. Polym..

[23] Kong X. (2012). Simultaneous determination of degree of deacetylation, degree of substitution and distribution fraction of -COONa in carboxymethyl chitosan by potentiometric titration. Carbohydr. Polym..

[24] Chen S.C., Wu Y.C., Mi F.L., Lin Y.H., Yu L.C., Sung H.W. (2004). A novel pH-sensitive hydrogel composed of N,O-carboxymethyl chitosan and alginate cross-linked by genipin for protein drug delivery. J. Control. Release.

[25] Tzaneva D., Simitchiev A., Petkova N., Nenov V., Stoyanova A., Denev P. (2017). Synthesis of carboxymethyl chitosan and its rheological behaviour in pharmaceutical and cosmetic emulsions. J. Appl. Pharm. Sci..

[26] Kumar S., Koh J. (2012). Physiochemical, optical and biological activity of chitosan-chromone derivative for biomedical applications. Int. J. Mol. Sci..

[27] Oluwasina O.O., Olagboye A.S., Boboye A., Hassan F.G. (2017). Carboxymethyl chitosan zinc supplement: Preparation, physicochemical, and preliminary antimicrobial analysis. Cogent Chem..

[28] Chandra Dey S., Al-Amin M., Ur Rashid T., Zakir Sultan M., Ashaduzzaman M., Sarker M., Md Shamsuddin S. (2016). Preparation, Characterization and Performance Evaluation of Chitosan As an Adsorbent for Remazol Red. Int. J. Latest Res. Eng. Technol..

[29] Corazzari I., Nisticò R., Turci F., Faga M.G., Franzoso F., Tabasso S., Magnacca G. (2015). Advanced physico-chemical characterization of chitosan by means of TGA coupled on-line with FTIR and GCMS: Thermal degradation and water adsorption capacity. Polym. Degrad. Stab..

[30] Lim B.Y., Poh C.S., Voon C.H., Salmah H. (2015). Rheological and Thermal Study of Chitosan Filled Thermoplastic Elastomer Composites. Appl. Mech. Mater..

[31] Ge H., Wang S. (2014). Thermal preparation of chitosan-acrylic acid superabsorbent: Optimization, characteristic and water absorbency. Carbohydr. Polym..

[32] Camilo P.-C., Bolanos G. (2019). Solubility of chitosan in aqueous acetic acid and pressurized carbon dioxide-water: Experimental equilibrium and solubilization kinetics. J. Supercrit. Fluids.

[33] Bidgoli H., Zamani A., Taherzadeh M.J. (2010). Effect of carboxymethylation conditions on the water-binding capacity of chitosan-based superabsorbents. Carbohydr. Res..

[34] Hubbe M.A. (2019). Why, After All, Are Chitosan Films Hydrophobic?. BioResources.

[35] Karava A., Lazaridou M., Nanaki S., Michailidou G., Christodoulou E., Kostoglou M., Iatrou H., Bikiaris D.N. (2020). Chitosan Derivatives with Mucoadhesive and Antimicrobial Properties for Simultaneous Nanoencapsulation and Extended Ocular Release Formulations of Dexamethasone and Chloramphenicol Drugs. Pharmaceutics.

[36] Bikiaris N.D., Michailidou G., Lazaridou M., Christodoulou E., Gounari E., Ofrydopoulou A., Lambropoulou D.A., Vergkizi-Nikolakaki S., Lykidou S., Nikolaidis N. (2020). Innovative skin product emulsions with enhanced antioxidant, antimicrobial and UV protection properties containing nanoparticles of pure and modified Chitosan with encapsulated fresh pomegranate juice. Polymers.

[37] Fathi M., Samadi M., Rostami H., Parastouei K. (2021). Encapsulation of ginger essential oil in chitosan-based microparticles with improved biological activity and controlled release properties. J. Food Process. Preserv..

[38] Jamil B., Abbasi R., Abbasi S., Imran M., Khan S.U., Ihsan A., Javed S., Bokhari H. (2016). Encapsulation of cardamom essential oil in chitosan nano-composites: In-vitro efficacy on antibiotic-resistant bacterial pathogens and cytotoxicity studies. Front. Microbiol..

[39] Othman N., Masarudin M.J., Kuen C.Y., Dasuan N.A., Abdullah L.C., Jamil S.N.A.M. (2018). Synthesis and optimization of chitosan nanoparticles loaded with l-ascorbic acid and thymoquinone. Nanomaterials.

[40] Lu P.J., Fu W.E., Huang S.C., Lin C.Y., Ho M.L., Chen Y.P., Cheng H.F. (2018). Methodology for sample preparation and size measurement of commercial ZnO nanoparticles. J. Food Drug Anal..

[41] Bootz A., Vogel V., Schubert D., Kreuter J. (2004). Comparison of scanning electron microscopy, dynamic light scattering and analytical ultracentrifugation for the sizing of poly(butyl cyanoacrylate) nanoparticles. Eur. J. Pharm. Biopharm..

[42] Murugappan R., Karthikeyan M., Aravinth A., Alamelu M. (2012). Siderophore-mediated iron uptake promotes yeast-bacterial symbiosis. Appl. Biochem. Biotechnol..

[43] Papadimitriou S., Bikiaris D., Avgoustakis K., Karavas E., Georgarakis M. (2008). Chitosan nanoparticles loaded with dorzolamide and pramipexole. Carbohydr. Polym..

[44] Zhihui J., Chun Y., Fangnan Z., Xiaolian C., Yuhi L., Huiping X. (2020). One-Step Reinforcement and Deacidification of Paper. Coatings.

[45] Bhujbal S.V., Mitra B., Jain U., Gong Y., Agrawal A., Karki S., Taylor L.S., Kumar S., Zhou Q.T. (2021). Pharmaceutical amorphous solid dispersion: A review of manufacturing strategies. Acta Pharm. Sin. B.

[46] Wesseling P., Ko B.C., Lewandowski J.J. (2003). Quantitative evaluation of α-Al nano-particles in amorphous Al87Ni7Gd6—Comparison of XRD, DSC, and TEM. Scr. Mater..

[47] Mattos A.L.C., Constantino V.R.L., de Couto R.A.A., Pinto D.M.L., Kaneko T.M., Espósito B.P. (2013). Desferrioxamine-cadmium as a “Trojan horse” for the delivery of Cd to bacteria and fungi. J. Trace Elem. Med. Biol..

[48] Ong S.G.M., Ming L.C., Lee K.S., Yuen K.H. (2016). Influence of the encapsulation efficiency and size of liposome on the oral bioavailability of griseofulvin-loaded liposomes. Pharmaceutics.

[49] Qayoom A., Aneesha V.A., Anagha S., Dar J.A., Kumar P., Kumar D. (2019). Lecithin-based deferoxamine nanoparticles accelerated cutaneous wound healing in diabetic rats. Eur. J. Pharmacol..

[50] Nieves I., Galbis E., Concepción V., De-Paz M.-V., Galbis J.A. (2018). Reversible pH-Sensitive Chitosan-Based Hydrogels.Influence of Dispersion Composition on RheologicalProperties and Sustained Drug Delivery. Polymers.

[51] Zha S., Utomo Y.K.S., Yang L., Liang G., Liu W. (2022). Mechanic-Driven Biodegradable Polyglycolic Acid/Silk Fibroin Nanofibrous Scaffolds Containing Deferoxamine Accelerate Diabetic Wound Healing. Pharmaceutics.

[52] Yao Q., Liu Y., Selvaratnam B., Koodali R.T., Sun H. (2018). Mesoporous Silicate Nanoparticles/3D Nanofibrous Scaffold- mediated Dual-drug Delivery for Bone Tissue Engineering. J. Control. Release.

[53] Farr A.C., Xiong M.P. (2021). Challenges and Opportunities of Deferoxamine Delivery for Treatment of Alzheimer’s Disease, Parkinson’s Disease, and Intracerebral Hemorrhage. Mol. Pharm..

[54] Ran Q., Yu Y., Chen W., Shen X., Mu C., Yuan Z., Tao B., Hu Y., Yang W., Cai K. (2018). Deferoxamine loaded titania nanotubes substrates regulate osteogenic and angiogenic differentiation of MSCs via activation of HIF-1α signaling. Mater. Sci. Eng. C.

[55] Marzban A., Akbarzadeh A., Ardestani M.S., Ardestani F., Akbari M. (2018). Synthesis of nano-niosomal deferoxamine and evaluation of its functional characteristics to apply as an iron-chelating agent. Can. J. Chem. Eng..

[56] Baghel S., Cathcart H., O’Reilly N.J. (2016). Polymeric Amorphous Solid Dispersions: A Review of Amorphization, Crystallization, Stabilization, Solid-State Characterization, and Aqueous Solubilization of Biopharmaceutical Classification System Class II Drugs. J. Pharm. Sci..

[57] Strober W. (2015). Trypan Blue Exclusion Test of Cell Viability. Curr. Protoc. Immunol..

[58] Saletta F., Rahmanto Y.S., Siafakas A.R., Richardson D.R. (2011). Cellular iron depletion and the mechanisms involved in the iron-dependent regulation of the growth arrest and DNA damage family of genes. J. Biol. Chem..

[59] You L., Wang J., Liu T., Zhang Y., Han X., Wang T., Guo S., Dong T., Xu J., Anderson G.J. (2018). Targeted Brain Delivery of Rabies Virus Glycoprotein 29-Modified Deferoxamine-Loaded Nanoparticles Reverses Functional Deficits in Parkinsonian Mice. ACS Nano.

[60] Katsarou A., Gkouvatsos K., Fillebeen C., Pantopoulos K. (2021). Tissue-Specific Regulation of Ferroportin in Wild-Type and Hjv-/- Mice Following Dietary Iron Manipulations. Hepatol. Commun..

[61] Charlebois E., Pantopoulos K. (2021). Iron overload inhibits BMP/SMAD and IL-6/STAT3 signaling to hepcidin in cultured hepatocytes. PLoS ONE.

